# Innate Immune and Inflammatory Responses to Respiratory Viruses

**DOI:** 10.1155/2019/3146065

**Published:** 2019-04-14

**Authors:** Shitao Li, Bishi Fu, Chetan D. Meshram

**Affiliations:** ^1^Department of Physiological Sciences, Center for Veterinary Health Sciences, Oklahoma State University, Stillwater, OK, USA; ^2^Medical Research Institute, School of Medicine, Wuhan University, Wuhan, China; ^3^Department of Microbiology, University of Alabama at Birmingham, Birmingham, AL, USA

Respiratory viruses (RVs), including but not limited to influenza virus, respiratory syncytial virus, coronavirus, rhinovirus, parainfluenza virus, adenovirus, and human metapneumovirus, can lead to severe diseases including bronchiolitis and pneumonia or/and to exacerbations of asthma and chronic obstructive pulmonary disease. Lung airway epithelial cells and mucosal immune cells are the primary cells for RVs. Following viral infection, these cells generate a range of mediators, including type I interferon (IFN), proinflammatory cytokines, and chemokines, which not only have pivotal roles in virus control but also determine the development of inflammation and disease. Given that vaccines are not available for RVs other than influenza virus, there is a critical need for the understanding of the interactions between RV and host innate immunity and the development of effective therapies limiting the severity of inflammation caused by RV infection.

This special issue presents five original research articles on host immune responses to different RVs, including influenza A virus (IAV), adenovirus, herpesvirus, and rhinovirus. Human adenovirus causes the most community-acquired pneumonia in infants and children with significant morbidity and mortality. Viruses are known to modulate host microRNAs (miRNAs), which are critical for viral replication and host immune response. However, the profiling of miRNA in children infected with adenovirus has not been reported yet. In this issue, F. Huang et al. examined the miRNA expression in the whole blood of adenovirus-infected pneumonia children and healthy controls by RNA sequencing. Their studies showed a distinct miRNA expression profile in adenovirus-infected children highlighted with the top three unregulated miRNAs (hsa-miR-127-3p, hsa-miR-493-5p, and hsa-miR-409-3p). Analysis of the host target genes of the microRNAs revealed that most target genes are involved in the MAPK signaling pathway and innate immune response. The microRNA profiling will not only help understand the role of miRNAs in modulating the host response to adenovirus infection but also provide potential biomarkers for adenovirus-infected pneumonia.

It has been well established that retinoic acid-inducible gene I (RIG-I) is a cytosolic sensor for RNA viruses, which binds viral RNAs, such as double-stranded RNA and 5′-triphosphate RNA. Engagement of viral RNA activates RIG-I, which initiates the MAVS-TBK1-IRF3 signaling cascade and induces type I IFN expression, thereby limiting the spread of infection. W. Wu et al. presented multiple lines of evidence that deficiency of RIG-I or MAVS neither resulted in higher mortality nor reduced IAV-induced cytokine responses in mice. RIG-I knockout mice displayed comparable lung inflammation as wild-type mice after influenza infection. RNA sequencing further demonstrated that both RIG-I wild-type and knockout mice exhibited comparable antiviral and inflammatory responses. As influenza activates multiple innate immune signaling pathways, such as TLR7 and NOD2, W. Wu et al. proposed a novel model that RIG-I serves as the primary PRR for IAV while TLR3, NOD2, MDA5, and TLR7 serve as the alternate PRRs for generating an innate response to IAV.

Acute exacerbation of idiopathic pulmonary fibrosis (AE-IPF) is a diagnosis of IPF with acute worsening of dyspnea in the preceding month and associated with high mortality. Whether pathogens can trigger AE-IPF is unknown. D. Weng et al. investigated the role of infection in AE-IPF by examining the changes in pathogen involvement during AE-IPF. They recruited 170 IPF patients (48 AE-IPF, 122 stable) and 70 controls. Their studies showed that the antiviral/bacterial IgM was higher in IPF vs. controls and in AE-IPF vs. stable IPF. Fifty-seven different viruses were detected in nasopharyngeal swabs of AE-IPF patients. AE-IPF also displayed abnormally activated inflammatory cytokines.

Recent studies show that CD4+ stem cell-like memory T cells (TSCMs) are a distinct memory T cell subset and preferentially reside in the bone marrow (BM). However, the existence and function of CD4+ TSCMs in a mouse, especially at the anatomical site of CD4+ TSCMs, were not well characterized. In this special topic issue, K. Wu et al. provided evidence that the BM acts as a hub for the relocation of most of antigen-specific CD4+ TSCMs. Furthermore, BM-resident TSCMs showed higher activity in inducing antibodies against influenza infection when compared with the spleen-resident TSCMs in mice. These findings may provide insights for future implications of immunotherapy against influenza.

Bovine herpesvirus type 1 (BoHV-1) infection causes inflammation in the respiratory tract and reproductive system in cattle of all ages and breeds. How BoHV-1 infection leads to inflammation is not well elucidated. X. Fu et al. found that BoHV-1 infection induces overproduction of reactive oxidative species (ROS), which are inflammatory mediators. Their studies further showed that interruption of the mitochondrial respiratory chain (RC) complexes by different chemicals reduced virus productive infection, suggesting that the integrity of RC complexes is critical for BoHV-1 replication. The virus infection significantly regulated the expression of various genes involved in the mitochondrial respiratory chain, antioxidant enzymes, and mitochondrial biogenesis-related signalings, such as MTCO1 SOD1/2, TFAM, and NRF1/2. These findings will advance the understanding of the mechanisms of BoHV-1 infection-induced ROS production and mitochondrial damage.

Overall, these original research articles will improve our knowledge of immune responses to RV infection, provide insights into the future design of effective antivirals, and pave the avenues for a rational basis for the development of potential therapeutic strategies.

